# Supplementary material for: Content-rich biological network constructed by mining PubMed abstracts
Source: BMC Bioinformatics. 2004 Oct 8;5:147. doi: 10.1186/1471-2105-5-147 (PMC528731; doi:10.1186/1471-2105-5-147)
Supplement: Additional File 2 — The original results of the above study (non-essential files are deleted to keep the file size under the limit set by BMC bioinformatics). [file 1471-2105-5-147-S2.bz2 › chilibotAdditionalFile2/dip20/548ID8872471E10901/html/AR_GAPD.html]

 


 **AR** and **GAPD** 
  
Found 11 abstracts in PubMed, retrieved 11.  
 

 What does Google say? 
 PDF only 
| .edu only 

---

**Interactive relationship** (e.g. stimulation, inhibition, etc)

**Neutral relationship**- The androgen receptor  [ **AR** ]  interacts with GAPDH  [ **GAPD** ]  both in the yeast two hybrid system and in vitro.  Ref: 8872471 Hum Mol Genet, 1996
- **AR**  transcript ranged from 302 to 1440 copies per 10 5 GAPDH  [ **GAPD** ]  copies and from 257 to 3223 copies per 10 5 GAPDH  [ **GAPD** ]  copies in the epithelium and stroma, respectively.  Ref: 11911259 Anticancer Res< MedlineTA>Anticancer Res, 1996

**Non-interactive relationship** (e.g. studied together, co-existance, homology, etc.)

- The binding of both ataxin 1 and the androgen receptor  [ **AR** ]  to GAPDH  [ **GAPD** ]  does not vary with the length of the polyglutamine tract.  Ref: 8872471 Hum Mol Genet, 1996
- In contrast, ARG and HB EGF mRNAs showed a coordinate pattern of expression in 7 10 specimens that was distinct from all other growth factor or receptor genes examined and from mRNAs for prostate specific antigen, the androgen receptor  [ **AR** ]  and GAPDH  [ **GAPD** ] , a house keeping enzyme.  Ref: 10579352 Endocrinology, 1999
- Mean data on lenticular enzyme activities GPX, G6PH, GAPDH  [ **GAPD** ] , ALD,  **AR** , LDH, PFK and SDH as well as measurements of GSH GSSG, ATP, ADP, AMP, Gluc, Fruc, Sorb, G6P and F6P do not indicate changes which may directly lead to lens opacifications.  Ref: 2151608 Lens Eye Toxic Res, 1990
- To investigate if GAPDH  [ **GAPD** ]  might interact with other glutamine repeat containing proteins involved in neurodegenerative disorders, we tested its binding to the androgen receptor  [ **AR** ]  which is mutated in spinobulbar muscular atrophy.  Ref: 8872471 Hum Mol Genet, 1996
- We have analyzed the expression of androgen receptor  [ **AR** ]   **AR** , progesterone receptor PR, estrogen receptor ER, clusterin CLU, complement C3 C3, and GAPDH  [ **GAPD** ]  mRNA in the rat uterus following oral administration of ethinylestradiol EE, bisphenol A BPA, o, p DDT DDT, p tert octylphenol OCT and daidzein DAI.  Ref: 10822019 J Steroid Biochem Mol Biol, 2000
- Inter and intra assay variability of gene quantitation was reflected by mean values of the variance coefficients of Ct of 4.84 1.13% and 2.73 0.39% or 3.32 1.03% and 2.21 0.24% corresponding to percent variances of copy numbers of 83.07 12.72% and 34.45 9.03% or 47.40 8.59% and 23.83 3.16% for human beta3  **AR**  and GAPDH  [ **GAPD** ]  genes, respectively.  Ref: 11735259 J Mol Cell Cardiol, 2001
- Primers and probes were designed to amplify the three subtypes of human beta adrenoceptors beta1, beta2 and beta3  **AR** , as well as reference genes such as glyceraldehyde 3 phosphate dehydrogenase  [ **GAPD** ]  GAPDH  [ **GAPD** ] , Hypoxanthine guanine phosphoribosyltransferase HPRT, and the oncogene ABL by real time RT PCR.  Ref: 11735259 J Mol Cell Cardiol, 2001
- On the other hand, the gene expression of alpha1b  **AR**  subtype did not change over the different stages in relation to that of a housekeeping gene, glyceraldehyde 3 phosphate dehydrogenase  [ **GAPD** ] , and to total  **AR** .  Ref: 11369312 Arch Oral Biol, 2001
